# Caught in the middle: a thematic analysis of the experiences of Korean-Canadian caregiver-employees in the greater Toronto and Hamilton area

**DOI:** 10.1186/s12889-021-11812-7

**Published:** 2021-09-23

**Authors:** Joonsoo S. Lyeo, Allison Williams

**Affiliations:** 1 Health Sciences Program, McMaster University, Hamilton, ON Canada; 2 3002 Preserve Dr.,, Oakville, Canada; 3 School of Earth, Environment & Society, McMaster University, Hamilton, ON Canada

**Keywords:** Ageing society, Canada, Caregiving, Elderly Korean immigrants, Work

## Abstract

**Background:**

The objective of this study was to investigate the experiences of caregiver-employees (CEs) from the Korean-Canadian community in the Greater Toronto and Hamilton Area.

**Methods:**

Nine participants were recruited and invited to partake in data collection, which consisted of the completion of a sociodemographic questionnaire as well as a qualitative, semi-structured interview. The interview transcripts were thematically analyzed.

**Result:**

The thematic analysis revealed four primary themes, each of which had three sub-themes. The four primary themes are:: (i) tensions, (ii) adaptations to the dual role of being a CE, (iii) coping mechanisms, and (iv) desired changes to the status quo.

**Conclusion:**

The result of this study suggest that Korean-Canadian CEs, as a consequence of their position at the convergence of Korean and Western cultural values, would be best supported through the provision of culturally sensitive supports and greater workplace accommodation.

## Introduction

Canada, alongside most other industrialized countries, has been experiencing a significant demographic shift [1]. As indicated by the results of the 2016 Canadian census, longer life expectancies and falling fertility rates have contributed to the aging of the national population [2]. The same census revealed that, for the first time in the country’s history, the share of seniors aged 65 years or older have superseded the share of children aged 14 years or younger [2]. Population estimates from 2019 indicate that this gap continues to widen, with seniors now comprising over 17.5% of the population and children accounting for less than 16.0%. Some projections predict that by 2031, the share of seniors will increase to as much as 22.7% [3].

Canada’s aging population is predicted to impact the quantity and quality of health, social, and long-term care services for seniors [1]. The anticipated increase in demand for such programs has called into question the ability of Canada’s formal healthcare services to cope without undergoing major reform [4]. As a result, it is important to consider the role of informal sources of long-term care, such as family caregivers. In 2012, an estimated 8 million Canadians were actively providing informal care and support for friends, spouses, parents, and other family members unable to independently take care of themselves [5]. Family caregivers provide an important safety net for many seniors with chronic health issues, and provide significant cost savings associated with formal healthcare services [5].

The growing number of seniors has been accompanied by decreases in the share of working-age adults in the national population [1]. Population projections released by the Ontario Ministry of Finance predict that the provincial working-age population, defined as those between the ages of 15 to 64 years, will fall from 67.2% in 2018 to 61.9% in 2046 [6]. The decline of the working-age population, coupled with a growing demand for long-term care services, may increase the likelihood of more individuals taking on the dual role of caregiver-employees (CEs), defined as individuals who provide unpaid care to a dependent while simultaneously engaging in paid employment [7]. Literature suggests that CEs often face unique challenges when attempting to manage the simultaneous responsibilities of providing unpaid care and carrying out paid work [8]. Some challenges faced by CEs include: higher levels of stress, detrimental physical and mental health outcomes, and emotional fatigue [8].

This study is concerned with the experiences of CEs of Korean-Canadian heritage. ‘Hyodo’ refers to a traditional Korean cultural value analogous to the Confucian tenet of filial piety [9]. It states that grown children have a responsibility to care for their parents, often as reciprocation for the care that they had received in their youth [9]. This value is not only adhered to by Koreans residing in their cultural homeland, but is followed by some Korean immigrants living abroad [9]. Korean immigrants in Canada thus lie at the confluence of traditional Korean cultural values - which tend to place a greater emphasis on familial obligations, and Western cultural values - which tend to place a greater emphasis on individualistic lifestyles [10]. As a result, the Korean diaspora in Canada, which consisted of 198,210 individuals in 2016, may provide insight into a culturally unique perspective on caregiving [11]. Although the vast majority of this community is likely of South Korean descent, there is a lack of data concerning the national origins of Korean-Canadians [12]. It should be acknowledged that the Korean-Canadian community may not necessarily be homogenous in this regard; the lack of data makes it difficult to distinguish South Korean immigrants from ethnic Koreans of other national origins, including North Koreans, Joseon, Koryo-Saram, etc. For context, it should be noted that the term ‘Joseon’ refers to a self-identification used by some ethnic Koreans residing in Japan, while the term ‘Koryo-Saram’ refers to a similar term used by some ethnic Koreans residing in the post-Soviet states of Central Asia [13].

There is a lack of research on the experiences of CEs in the Korean-Canadian community; however, research does exist on the experiences of CEs in the broader Korean immigrant diaspora. Much of this literature was produced in the United States, reflecting the experiences of Korean-American CEs. One study by Lee et al. concluded that filial expectations and interpersonal conflict often shaped the perspectives of American-born Korean caregivers [14]. Another study by Kim and Theis also identified the importance of filial piety, as well as the prohibitive effect of language barriers, for Korean-American family caregivers [15]. As these findings were specific to Korean-American CEs, assessing their relevance to the Korean-Canadian community provides a comparative opportunity. Further, identifying the particular challenges faced by Korean-Canadian CEs, as noted by members of the community themselves, provides the opportunity to develop culturally sensitive supports aimed at reducing the burdens they face. This study aims to understand the perspectives of Korean-Canadian CEs in the Greater Toronto and Hamilton Area (GTHA), with respect to the simultaneous management of their caregiving duties and their paid employment.

## Subjects and methods

Ethics approval for this study was obtained from the McMaster Research Ethics Board (MREB#: 2175). Korean-Canadian CEs were recruited from the GTHA using the following strategies: online advertisements posted on social media, brochures distributed at community centres, posters placed at ethnic supermarkets, and presentations given to the members of adult day programs. Prospective participants were required to complete a screening questionnaire, which assessed whether they fit the selection criteria for inclusion in the study. The selection criteria were applied to all prospective participants to ensure that they: (i) were of Korean heritage, (ii) lived in Canada, and (iii) had provided care for a dependent with chronic health issues while simultaneously employed in the labour market. A total of 9 participants, consisting of 6 women and 3 men, completed the screening questionnaire and were subsequently found to be eligible to participate in the study. These participants were interviewed about their experiences as CEs. For additional information on the sociodemographic characteristics of the study participants, refer to Tables 1 and 2.

**Table 1:** Sociodemographic Characteristics of Caregiver-Employees (CEs) (*n* = 9)

Name	Caregiver Status	Caregiving Length	Relationship with Care Recipient	Age	Gender	Marital Status	Residence in Canada	Income	Education	Religion
Caregiver 1	Current	4–6 years	Daughter	56–65 years	Female	Married	> 16 years	$40,001–60,000	Bachelor’s	Protestant
Caregiver 2	Current	4–6 years	Son	36–45 years	Male	Married	> 16 years	>$60,000	Bachelor’s	Catholic
Caregiver 3	Current	4–6 years	Wife	66–75 years	Female	Married	> 16 years	<$20,000	High School	Protestant
Caregiver 4	Current	> 10 years	Daughter	46–55 years	Female	Married	> 16 years	>$60,000	Bachelor’s	Protestant
Caregiver 5	Current	4–6 years	Son	26–35 years	Male	Domestic Part.	> 16 years	>$60,000	Bachelor’s	None
Caregiver 6	Current	1–3 years	Daughter	56–65 years	Female	Married	> 16 years	$20,000–40,000	Some College	Protestant
Caregiver 7	Current	> 10 years	Daughter	26–35 years	Female	Domestic Part.	> 16 years	>$60,000	Bachelor’s	None
Caregiver 8	Former	< 1 year	Son	> 75 years	Male	Married	> 16 years	<$20,000	Bachelor’s	Protestant
Caregiver 9	Current	< 1 year	Daughter	26–35 years	Female	Single	> 16 years	>$60,000	Master’s	None

**Table 2:** Aggregate Sociodemographic Characteristics of Caregiver-Employees (CEs)

Variable	n	Variable	n
Caregiver Status		Marital Status	
Current	8	Married	6
Former	1	Domestic Partnership	2
		Divorced/Separated	0
		Single	1
Caregiving Length		Residence in Canada	
< 1 year	2	< 5 years	0
1–3 years	1	6–10 years	0
4–6 years	4	11–15 years	0
7–9 years	0	> 16 years	16
> 10 years	2		
Relationship with Care Recipient		Annual Household Income	
Child	8	<$20,000	2
Sibling	0	$20,000–$40,000	1
Parent	0	$40,001–$60,000	1
Spouse	1	>$60,000	5
Friend	0		
Other	0		
Age		Highest Degree Completed	
< 15 years	0	Less than highschool	0
16–25 years	0	Highschool diploma	1
26–35 years	3	Some college, no degree	1
36–45 years	1	Associate’s degree	0
46–55 years	1	Bachelor’s degree	6
56–65 years	2	Master’s degree	1
66–75 years	1	Professional degree	0
> 75 years	1	Doctorate	0
Gender Identity		Religious Denomination	
Male	3	Buddhism	0
Female	6	Catholicism	1
Other	0	Protestantism	5
		Folk Religion	0
		No Religion	0
		Other	0

After completing the screening questionnaire, participants who fit the selection criteria were invited to select a time and location, at their convenience, for data collection. Upon meeting with the researcher, participants were assured that all information provided during data collection would remain anonymous and confidential. The researcher also explained that participants were permitted to decline to answer any questions asked, and had the right to retroactively withdraw any information from the study. All participants provided their written and verbal informed consent prior to data collection. This was documented through the use of oral informed consent logs and signed letters of informed consent.

There were two phases of data collection. In the first phase, participants responded to a sociodemographic questionnaire consisting of ten close-ended questions. In the second phase, participants attended an interview which, based on the availability of each participant, lasted between 0.5 to 1.5 h. A semi-structured, biographical approach was used for the interviews. In the interviews, participants were encouraged to lead the conversation while the researcher provided as few prompts as possible. In the instances where prompts were provided, they were posed as open-ended questions which explored the experiences of participants and the stressors they faced as a result of their dual role as CEs. Listed below are several examples of open-ended questions asked during the interview:

- Please tell me about your experience caring for your family member/friend/etc.
- How has your daily routine changed since taking on the role of a caregiver?
- Have there ever been any conflicts between you and the recipient of your care?

It should be noted that only two people were present at each interview: the researcher conducting the interview, and the participant being interviewed. To compensate participants for their time, each received a $25 coffee shop gift card.

Interviews were audio-recorded whenever informed consent was received (*n* = 4). In such cases, the audio recordings were used to transcribe the interviews verbatim. When informed consent for audio recording was not received (*n* = 5), the researcher instead received informed consent to manually transcribe the interviews verbatim as they occurred. In such cases, the researcher transcribed the interview themselves, during and immediately following the interview. As the interviews were conducted in the preferred language of the participants, all interview transcripts were translated into English whenever necessary (*n* = 2). The researcher was conversationally fluent in both English and Korean. It should also be noted that, when possible, follow-up interviews were conducted to verify the accuracy of the data collected from the study participants.

A thematic approach was used to approach and analyze the data [16]. Interview transcripts were hand-coded, which allowed for the organization of recurring ideas into broader categories. Each interview transcript was repeatedly analyzed until no additional categories could be identified by the researcher. The resulting categories were then consolidated and reduced to a manageable number. The categories created through this process served as the basis for the study themes. The items organized under each theme were further categorized into sub-themes, based on recurring elements within the broader theme. The themes and sub-themes identified in this manner were interpreted to reflect the key ideas underlying the responses of the participants in their interviews.

## Results

A thematic analysis of the interviews revealed four themes underlying the experiences of Korean-Canadian CEs: (i) tensions, (ii) adaptations to the dual role of being a CE, (iii) coping mechanisms, and (iv) desired changes to the status quo. Each of the primary themes has been further subdivided into three sub-themes. In this section, each primary theme and associated sub-themes will be discussed in detail.

### Theme 1: tensions

This theme addresses the internal and external conflicts generated by each participant’s caregiving duties. The sub-themes address how engaging in these duties has caused participants to: (i) experience emotional distress; (ii) neglect their own needs; and (iii) strain their social networks.

#### Emotional distress

All 9 participants reportedly experienced some form of emotional distress due to their caregiving duties. Participants typically described their emotional distress as consisting of feelings of anxiety, depression, and stress. One participant (*Caregiver 1*), explained:

“Shortly after leaving work, I fell into a depressed state. I watched my mother fall deeper into dementia. Without my work to distract me, I had no choice but to confront my feelings. It made me question the value of life. I saw my mother losing her mind to dementia and it made me think: is this what I have to look forward to? What’s the point?”

Participants stated that emotional distress contributed to feelings of burnout, compassion fatigue, and exhaustion with respect to their caregiving duties. Participants explained that this emotional distress not only had a negative impact on their caregiving, but also on other elements of their day-to-day life, such as their work and leisure. For instance, *Caregiver 4* described the impact of compassion fatigue on her workplace interactions.

“There are definitely days when I come into work more exhausted. I worry of becoming emotionally numb to what I do, which would be a real big problem, since I work in healthcare …. I don’t want to be this numb and unfeeling husk that treats people like quotas. I don’t want my sympathy to burn out. I worry that if I don’t manage to catch a breath every now and then, and get a break, then that’s the direction I’m heading.”

#### Neglect of own needs

Five participants felt that they had to neglect their own physical and psychological needs in order to fulfill their caregiving duties. Some participants explained that this was because they did not have the time or financial resources to commit to both sets of responsibilities. For instance, *Caregiver 3* stated:

“It’s hard to pick a single thing about it, because it feels like I’m always tired. There’s no time for a break, because there is always something that needs to be done. It can be frustrating.”

When presented with the option of caring for their dependents or attending to their own needs, they were more CEs willing to make sacrifices to the latter in favour of the former. In other cases, participants stated that the neglect of their own needs was the result of their inability to distance themselves, both physically and emotionally, from their caregiving. This idea was discussed in the following excerpt from *Caregiver 6*:

“It can be so hard to get time off. Even when I do have some time to myself, I always find myself worrying about my mother and how she’s doing. Now it feels like a large part of my life revolves around her, even when she’s not actually there next to me.”

#### Straining of social networks

Six participants stated that their caregiving duties have become a source of conflict in their personal relationships. In some instances, this conflict occurred when care recipients, especially parents, moved into the same household as their caregivers. In such cases, the resulting changes to domestic life were often significant. For instance, participants reported that after their care recipients moved in with them, members of their household often had to make changes to elements of their day-to-day life, including their sleep regimens, the distribution of chores, and household expenditures. These changes to the status quo of domestic life often manifested as conflicts between caregivers and other residents of the same household. *Caregiver 1* explained how such tensions have strained her relationship with her son:

“My son loves his grandmother, I know he does, but I understand why it is hard for him to be living with her when she is in this state. I remember my son and I had a fight where he mentioned he was too embarrassed to bring friends over because he didn’t want them to see his grandmother. I was furious and just so shocked that he would even say that.”

Participants also faced, or at least anticipated, disagreements within their family network regarding their approach to their caregiving duties. It was reported that such tensions were the result of the discrepancies between two differing perspectives on caregiving: a hands-on approach associated with traditional Korean culture, and a relatively hands-off approach associated with Canadian or Western culture. This discrepancy is highlighted in the following quote from *Caregiver 8*, regarding his decision to place his mother in a nursing home:

“My brother would be furious with me if he found out I was asking for outside help. He would think I am failing as a son …. He was always rather traditional, more than me. I think it is because I live in Canada while he has always lived in Korea. He would think that I should be ashamed for being unable to give my mother a good life on my own.”

In summary, participants report that their caregiving was often a source of conflict, both at an interpersonal and intrapersonal level. The resulting tensions were often catalyzed by the filial expectations associated with Korean cultural values. For instance, participants often prioritized their caregiving duties over most of their own needs. This placed further strain on participants in their role as CEs. Furthermore, as seen in the third sub-theme, the differences between Western and Korean attitudes on care were also a source of conflict. This conflict was typically seen between individuals with different levels of attachment to traditional Korean cultural values.

### Theme 2: adaptations to the dual role of a caregiver-employee

This theme addresses the strategies participants used to balance their dual role as both caregivers and employees, including the set of responsibilities carried out in either role. The sub-themes address how participants managed their work-life balance by: (i) decreasing the physical distance between their work and home, (ii) reducing their commitments to their paid employment, and (iii) seeking external support with caregiving duties.

#### Decreasing the physical distance between work and home

Three participants reduced the physical distance between their home and their workplace in order to better manage their work-life balance. This decision was often influenced by participants’ desire to reduce both their exhaustion as well as the time spent commuting, which they hoped would allow them to set aside more time and energy for their caregiving duties. For instance, *Caregiver 8* explained his decision to work from home:

“After my mother’s fall, I had to work from home. That was a bigger change than I would’ve thought. I thought working at the office was tiring because I would go from working at the office to coming home to take care of my mother. I was leaving work to come back to more work. It was too much and nothing was getting enough of my time.”

However, *Caregiver 8* explained that his decision to work from home has made it difficult for him to distinguish the distress resulting from his paid employment from the distress caused by his caregiving duties. He claimed that his inability to separate these sources of distress has in turn compounded the magnitude of distress he feels:

“After I started to work from home, it made me realize how good I had it. Before, I had a change of setting from the office to home. Then my house became my office, and I combined those two sets of responsibilities. It no longer felt like I was having to balance taking care of my mother and taking care of my work. It felt like I was doing both at once.”

#### Reducing commitments to paid employment

Eight participants have tried to prioritize their caregiving duties by reducing their commitments to their paid employment. Several different approaches were used to this end. For instance, after she began caregiving for her mother, *Caregiver 6* decided to step down from her full-time position to work part-time, accepting a decrease in salary in order to allocate more time to her caregiving:

“I am part of a cabin cleaning team, which I do part-time. I used to work full-time, which is what I did for the last 20 years, but within the last year or so, when I started caring for my mother, I decided it would be best to scale it back. I felt more comfortable that way, knowing that I could still get a comfortable salary, with my husband in his job still, while also having the time to come home and care for my mother for most of the day.”

In another instance, *Caregiver 1* explained that she had applied for short-term disability leave. She explained that after the first month of her disability leave, she faced pressure from her manager to return to work. She stated that her manager presented her with an ultimatum, asking her to either return to work or be let go. Viewing this incident as an indicator of the incompatibility of her caregiving and her paid employment, *Caregiver 1* eventually decided to quit altogether in order to focus on providing care for her mother:

“When my mother’s dementia started to get worse and more problematic, I had to quit work to take care of her. Well, I had short term leave as well, but it was only for a month. After the month was up, my manager told me that I’d either have to come back to work, or else they’d have to let me go. My company was not at all flexible, so I decided to go.”

*Seeking external support with caregiving*: Six participants have accessed external support services and programs, beyond their personal social network, to assist with their caregiving. Examples of external support accessed by participants include adult daycare centres, nursing homes, and psychotherapists. All participants reported that their care recipients were unable to communicate in English. As a result, participants made an effort to seek out Korean-language services and prioritized culturally sensitive programs. *Caregiver 3* explains her decision to bring her husband to a specific Korean-language adult day program, despite it being located two hours from her home, on a weekly basis:

“For a few hours every week, I can just relax and breathe. It’s so much less tiring, knowing that he is safe with people who know how to take care of him. And here there are people like him. Not just with similar conditions, but people who look like him, talk like him, eat like him. I think that makes him feel so much safer.”

In summary, participants struggled to simultaneously balance their caregiving with their paid employment. Participants often had to reduce their commitment to one set of responsibilities to dedicate more attention to the other. In other words, participants who reduced their commitments to their paid employment often did so with the intent of focusing on their caregiving. Conversely, those who reduced their caregiving commitments often did so with the intent of focusing on their paid employment.

### Theme 3: coping mechanisms

This theme addresses the strategies which participants used to manage the emotional distress they experienced as a result of their caregiving. The sub-themes address coping mechanisms commonly used by participants, including: (i) reminding oneself of their filial responsibility, (ii) seeking emotional support from social networks, and (iii) turning to religion.

#### Reminding oneself of their filial responsibility

Six participants reminded themselves of their filial responsibility to help manage the emotional distress associated with their caregiving duties. These participants made reference to their cultural upbringing, influenced by traditional Korean values, which emphasized the importance of fulfilling their filial obligations. In this regard, they justified the emotional distress that accompanied their caregiving as being necessary and part of their duty. For instance, *Caregiver 1* stated:

“We were raised on the same values that our parents were raised on, and for many of us, that’s our only earnest connection with Korean culture. The younger generation in Korea does not seem to care as much for upholding this tradition, where we, the children of our parents, turn back to take care of them when they need us.”

*Caregiver 2* described his caregiving as a necessary, reciprocal obligation to his mother:

“It’s a major motivation for me. What I mean is that when I get frustrated, I try to take a moment to step back and remind myself of how much my parents sacrificed for me and how much they cared for me as I was growing up.”

*Caregiver 8* echoes this sentiment in the following excerpt:

“It’s because she is my mom. When I got tired from caring for her, I would remind myself: if I don’t do this, who will? As her son, it is something that I had to do. It was more than just a responsibility, it was my everything.”

#### Seeking emotional support from social networks

Four participants confided in friends or family to manage the distress associated with their caregiving. In such instances, participants turned to their social networks for assistance and emotional support regarding the stressors resulting from their caregiving and their employment. Many participants were more comfortable accessing the informal support offered by their social networks as opposed to the support offered by formal institutions. Furthermore, participants were generally more comfortable confiding in those with similar experiences as CEs. For instance, *Caregiver 5* explained:

“It helps having a person that I can talk to about my parents and my concerns about them going into the future, someone who really gets it …. It’s a huge weight gone, having someone who knows what I’m thinking and can validate my concerns and thoughts.”

#### Turning to religion

Three participants coped with their emotional distress by turning to religion. These participants stated that their religious practices provided a source of comfort and hope, which alleviated some of the emotional distress they faced. These participants stated that praying allowed them to perceive their caregiving duties, as well as the health of their recipients, from a more comforting angle. For instance, *Caregiver 1* described how turning to religion has allowed her to rationalize her mother’s declining health:

“Going back to my faith really helped me get through the lowest points. I remember asking how God could let my mother fall to dementia when she had been nothing but kind her entire life, but later I came to see this as a sign. Caring for her made me more sympathetic to other people and their suffering.”

In summary, the coping mechanisms employed by participants often reflected some dimension of their cultural heritage. For instance, some participants stated that they would remind themselves of their filial responsibility when they felt overwhelmed by their caregiving duties. This cultural expectation not served as a source of motivation. Furthermore, religion was used as a coping mechanism by participants of Christian denominations. This use of religion often allowed participants to rationalize and accept that several stressors associated with their caregiving were beyond their locus of control.

### Theme 4: desired changes to the status quo

This theme addresses the systemic changes which participants believed would have a positive impact on their role as CEs. The desired changes can be grouped into three categories: (i) greater access to culturally sensitive support for their caregiving duties, (ii) greater access to financial support, and (iii) greater understanding from others.

#### Greater access to culturally sensitive support

Six participants were unsatisfied with their current level of access to services and programs offering culturally sensitive support with their caregiving duties. The necessity of such programs was emphasized by two points. As mentioned, all care recipients were reportedly unable to communicate in English. Secondly, 4 participants stated that although they were more comfortable communicating in English than their care recipients, they still had difficulty understanding English and would rather communicate in Korean whenever possible. These circumstances illustrate the importance of culturally sensitive programs in this study.

Some participants attributed this insufficient level of access to the lack of programs offering culturally sensitive care services. Furthermore, participants tended to question the quality of the few programs which do exist, many of which operate informally. *Caregiver 2* discussed both points in the following excerpt:

“It’s hard enough to find nursing programs with availabilities. Needing one that can provide services in Korean narrows our options. Then, of the nursing programs that qualify, we need to make sure they’re actually good. It feels like it’s downright impossible. There are a few informal programs we found, but they’re just so sketchy. I wouldn’t be surprised if they were unlicensed, you know, doing it under the table.”

Other participants explained that most programs offering culturally sensitive services seemed to be affiliated with local churches and religious organizations, which posed a barrier for those identifying as irreligious or from other religious denominations. *Caregiver 9*, who describes herself as an atheist, stated:

“Religion needs to be removed from healthcare organizations that want to serve the Korean-Canadian community in Toronto. Like that religious part and God was not really helpful to me, and was actually alienating.”

*Caregiver 9* identified a second barrier to accessing culturally sensitive support. She explained that when she first started caregiving for her mother, she struggled to find any information on the availability of culturally sensitive programs relevant to her needs. She explained that the lack of access to information on such programs was a significant source of stress, causing her to feel further isolated and burdened as a result of her situation:

“There’s no centralized way of getting information. It still runs how the Korean-Canadian community ran in the 70s and 60s, which is basically just word-of-mouth. Not even the internet wasn’t really helpful. All the websites were dead or gibberish. As someone who wasn’t involved in the Korean-Canadian community, it was impossible to find anything.”

#### Greater access to financial support

Three participants stated that they would like to reduce their work commitments to focus on their caregiving duties, but lacked the financial means to do so. These participants stated that receiving financial support would not only allow them to devote more time to their caregiving duties, but would also allow them to provide better quality care to their care recipients. These perspectives are discussed in the following excerpt by *Caregiver 4*:

“I don’t think I could afford quitting my job to focus on taking care of things at home. We live fairly comfortably now, but that’s with me bringing in most of the income. My husband also works, though I don’t think his income alone would be enough to support us if I quit my job to focus on my parents. Not to mention the cost of supporting our children and my parents. It’s scary, since if anything happens, a three month unpaid leave from work would be devastating. I don’t know what we could do on our own.”

#### Greater understanding from others

Seven participants stated that it would be easier for them to maintain their work-life balance if those around them were more understanding of their status as CEs. Some participants stated that they would want this greater understanding to come from their workplace, especially from their employers. For instance, *Caregiver 5* noted that his employer struggled to understand his situation:

“It’s like management is trying to make me feel guilty for taking time off to help my parents. I don’t like being guilted into picking one over the other, you know? My work or my parents …. I wish they tried to understand my situation better. I’m not trying to cheat the system. I’m not enjoying my days off. I’m going home to my dying mother.”

Other participants stated that they wished to receive a greater amount of understanding from their personal social networks. *Caregiver 7* explained how a lack of understanding made it difficult to confide in others, especially friends and family in the Korean-Canadian community, about the frustrations resulting from her caregiving:

“There is, I think, a cultural stance against asking for favours to avoid burdening them. The downside of this is that there is this stigma around even talking about the frustrations I face as a caregiver, so it can be hard to sit down with people I know in the community and say ‘you don’t need to do anything, I just need someone to listen.’”

In summary, participants proposed several changes to the status quo which they believed would alleviate some of the stressors they faced as CEs. Many of the changes proposed by participants reflected the differing expectations of Western and Korean cultural attitudes with respect to care. Participants felt that they were caught in the middle of these two perspectives, often wanting to increase their caregiving in accordance with Korean cultural values but being unable to do so due to societal constraints reflecting Western cultural values.

## Discussion

The study findings suggest that participants perceived their caregiving duties in a way that reflected their social and cultural heritage as part of the Korean-Canadian community. As all participants have resided in Canada for at least 16 years, it should come as no surprise that their perspectives on caregiving appeared to be a blend of Korean and Western cultural attitudes. Participants generally believed that Korean cultural attitudes were more family-oriented and placed a greater emphasis on one’s direct involvement in care provision. On the other hand, participants generally believed that Western cultural attitudes were focused on individual motivations, placing less emphasis on one’s direct involvement in care provision. The data indicate that the differing expectations of these cultural attitudes were often a source of conflict for participants. This conflict tended to take one of two forms: intrapersonal or interpersonal. In the context of this study, ‘intrapersonal conflicts’ refer to how a CE’s actions may have affected their own behaviour, whereas ‘interpersonal conflicts’ refer to how a CE’s actions may have affected another individual’s behaviour or vice versa [17].

With respect to intrapersonal conflicts, participants wished to dedicate more time and effort to their care provision but were unable to do so without reducing their current quality of life. This was due to social and economic stressors, including: the cost of living, a lack of workplace accommodations, and fear of judgement by supervisors and coworkers. Participants perceived these pressures to be common in Canadian society and believed them to be a reflection of Western cultural attitudes on care, characterized as having less of an expectation for individuals to directly care for family and friends. Participants believed that this particular perspective was unaccommodating given Korean cultural attitudes, in which there was a greater expectation for individuals to directly care for family and friends. Participants noted that the aforementioned stressors made it difficult for them to increase their level of caregiving without compromising their economic, physical, and mental wellbeing. As a result of these constraints, the actual level of caregiving displayed by participants did not necessarily reflect their own beliefs and attitudes of what ought to be. This may mark a significant difference between the experiences of Korean-Canadian CEs and Korean CEs residing in their cultural homeland.

With respect to interpersonal conflicts, younger participants - who typically described themselves as assimilated into Canadian society, faced tensions with members of the Korean-Canadian community who had a stronger adherence to traditional cultural values. These conflicts were the result of disagreements over the extent to which participants should directly involve themselves in the care received by their care recipients. While younger participants were typically willing to access external assistance in the form of interpreters, nursing homes, etc., these resources were viewed as being less acceptable by older members in the Korean-Canadian community. Similar attitudes have been documented among caregivers from other cultural groups emphasizing filial piety. A study by Donovan and Williams, revealed that Vietnamese-Canadian caregivers “did not believe that care recipients in hospitals and nursing homes would receive the level of care needed, especially compared with what they could provide at home” [18]. This statement mirrors the preference observed among older members of the Korean-Canadian community, in which formal caregiving was deemed to be less acceptable than informal caregiving.

Cultural differences also influenced the type of caregiving provided by participants. All but one of the participants stated they were providing care for their parents, who were described as comparatively less assimilated into Canadian society. As a result, participants often had to mediate between Korean and Canadian culture in their caregiving role. This was most notable in the context of language, as most care recipients were unable to communicate in English. Participants were responsible for scheduling appointments and speaking with medical staff, thereby allowing their care recipient to access formal healthcare services despite language barriers. Although care recipients heavily relied on their caregivers to access the Canadian healthcare system, there was sometimes a cultural divide between them. This was notable among Canadian-born participants who were caring for parents who had emigrated from Korea. These participants tended to be less comfortable communicating in Korean than English, casting doubt on their ability to relay healthcare information in a nuanced manner. Previous studies have documented the effects of language barriers on immigrant access to healthcare. For instance, a study by Sethi et al. (2017) found that language barriers were a major stressor for Mandarin-speaking CEs [19]. The study noted that Mandarin-speaking care recipients were often unable to independently access healthcare services outside of their respective communities due to language barriers, thus adding to the burden experienced by their caregivers. Similarly, another study by Sethi et al. (2018) also identified intergenerational differences as a source of stress and emotional conflict for CEs [20]. The intergenerational differences identified in Sethi et al’s (2018) study extended beyond the role of language, including factors such as food preparation, etc. [20]

Gender also had a significant role in shaping the caregiving experiences of participants. Of the 9 participants recruited into the study, 6 identified themselves as female and 3 identified themselves as male. There seemed to be a greater expectation for female children, as opposed to their male siblings, to take on the responsibility of caring for their parents. For instance, 3 of the 5 female participants with experience caregiving for a parent stated that they had male siblings, whereas the remaining 2 were the only children in the family. These participants shared the sentiment that the responsibility of caring for their parents “naturally fell to them” rather than their male siblings. Furthermore, 1 of these 3 participants stated that in addition to caring for her own mother, she was also expected to provide care for her husband’s parents. In contrast, all 3 male participants stated that they had male siblings, with 2 of them reporting that they were the eldest child. In the 1 remaining case, the participant in question shared his belief that it was unusual for him, as the youngest child, to be caring for his mother. It should also be noted that male participants were generally more hesitant, relative to their female counterparts, to reduce their commitments to their paid employment to focus on their caregiving. Similar findings were reported in a scoping review by Maynard et al., which proposed that this tendency may be a product of the gendered nature of the workforce [21]. It was stated that in “couple [s] faced with work–care conflicts, the partner earning less and with less job security will assume the caring role” which, in the context of the current gendered distribution of workforce participation and wage, increased the likelihood of women in a household sacrificing their employment [21].

Furthermore, prior studies have examined religious differences as a factor preventing immigrant caregivers from accessing support outside their respective communities [22]. However, there is a lack of literature examining religious differences as a factor preventing caregivers from accessing support within their communities. In response to the sociodemographic questionnaires issued as part of this study, 55.56% of participants identified themselves as Protestant, 11.11% as Catholic, and 33.33% as having no religion. The religious makeup of the study participants broadly reflects the data collected from the 2001 Canadian census, where 51% of Korean-Canadians identified themselves as Protestant, 25% as Catholic, and 20% as having no religion [23]. The dominance of Protestant Christians in the Korean-Canadian community is reflected by the affiliation of many culturally sensitive support programs in the GTHA with Protestant Christian churches and community centres. As mentioned, this particular observation was also noted by participants of the study. Non-religious participants reportedly felt alienated by the influence and incorporation of religion in the services provided by these programs. These participants stated that they would prefer to attend secular programs offering the same services, but were unable to do so because of their limited availability.

Participants also faced challenges commonly experienced by CEs in general, regardless of their social or cultural heritage. For instance, participants often struggled to simultaneously manage their responsibilities both as caregivers and as employees. Due to limitations in time, energy, and resources, participants often felt that they had to reduce their commitments to one of these areas of responsibility in order to allocate more attention to the other. When participants chose to distance themselves from their caregiving in order to focus on their paid employment, they often felt guilt, emotional distress, and judgment from their social networks. In contrast, when participants chose to distance themselves from their paid employment to focus on their caregiving, consequences included early retirement, extended leaves, and shifts from full-time to part-time work. Participants noted that their decision to choose one area of responsibility for the other tended to be triggered by a lack of accommodation from their workplaces, which offered them little flexibility and left them in precarious situations. This was especially problematic when participants encountered sudden and unpredictable events associated with caregiving, such as the onset of a medical emergency. Coupled with a lack of workplace accommodations, such events often caused participants to realize that they could not continue to manage both areas of responsibility at a sustainable level. These assessments are corroborated by conclusions drawn from prior literature. For instance, in a scoping review by Ireson et al., it was stated that a “lack of employer support has health and financial consequences for caregiver-employees, such as missed workdays, early retirements and reduced productivity” [24]. The authors also advocated for the implementation of policies which could render workplaces more accommodating to CEs by taking into account the unique challenges they face. A systematic literature review by Plöthner et al. identified many of the same factors when assessing challenges commonly encountered by CEs [25].

### Implications for research and practice

The study findings may be used to develop strategies aimed at supporting Korean-Canadian CEs. For instance, an effort could be made to increase the availability of care support programs, such as respite day and home care programs, offering services in both English and Korean. This may allow for culturally sensitive communication of information to caregivers and their care recipients.

It may also be beneficial to increase the availability of culturally sensitive services (e.g. patient navigators, translators, etc.) within the healthcare system, especially those catering to traditional Korean cultural attitudes emphasizing the role of caregivers in care provision. This could increase the degree to which Korean-Canadian CEs feel comfortable accessing formal support. Furthermore, efforts could be made to increase the availability of secular programs offering culturally sensitive caregiving support. This may reduce the degree of alienation felt by non-religious and non-Protestant members of the Korean-Canadian community.

Finally, strategies could be implemented to render workplaces more accommodating to the needs of employees who are simultaneously caregivers. This may include employers being exposed to informational resources explaining the challenges faced by CEs in general, as well as the challenges unique to CEs of a particular cultural heritage (see ghw.mcmaster.ca). This could promote a greater degree of understanding from employers with respect to the particular circumstances of their employees.

In future research on the experiences of CEs in the Korean diaspora, it may be valuable to see how the experiences of Korean immigrants living in other countries compare to the findings in this study. We anticipate that the study findings will be largely applicable for Korean CEs residing in countries with similar social, cultural, and economic contexts as Canada, such as Australia, as we have seen with the research from the United States. That being said, these findings may be less applicable for Korean CEs residing in developing, or least-developed economies with a non-Western cultural context.

### Limitations

The scope of this study is not without limitations. To begin, the external validity of this study, as with most qualitative research, may be undermined by its small sample size. The study findings may thus not be generalizable to Korean-Canadian CEs beyond the participants of this study. Furthermore, as indicated by the results of the sociodemographic questionnaires, all participants in the study have resided in Canada for at least 16 years. This may not be representative of the general Korean-Canadian community, considering how South Korea represented the tenth-largest source of immigration to Canada in 2016 alone [26]. It should also be noted that the overwhelming majority of participants reported that they were caring for parents, and thus may not represent the circumstances of Korean-Canadian CEs providing care for siblings, friends, etc. Moreover, as caregiving is a gendered experience, it should be acknowledged that all study participants who disclosed their gender identity and sexual orientation stated that they were cisgender and heterosexual respectively. The findings of this study may not reflect the needs and perspectives of individuals of non-binary gender identities and non-heterosexual orientations. In order to develop a greater and more relevant insight into the experiences of Korean-Canadian CEs, it would be beneficial for future studies to recruit a larger sample size of participants from a greater diversity of circumstances. Finally, while this study used a qualitative approach to obtain a nuanced understanding of the experiences and perspectives of Korean-Canadian CEs, future studies may use quantitative measures in addition to qualitative ones so as to gain a more precise understanding of their experiences and burdens.

## Conclusion

This study examined the perspectives of Korean-Canadian CEs regarding their dual role as both paid employees and informal caregivers. The findings suggest that in addition to the challenges commonly faced by CEs in general, CEs in the Korean-Canadian community tend to encounter challenges unique to their social and cultural heritage. The study findings have important implications for formal program planners in the Canadian healthcare system, as well as informal program planners in the Korean-Canadian community. As both communities seek to address the challenges of an aging population, further support should be extended to informal family caregivers. Programs aiming to support CEs of a particular social and cultural heritage must be cognizant of the unique challenges they face and, in so doing, provide them with the most effective and culturally sensitive solutions possible.

## Data Availability

The datasets used and analysed are available from the first author on reasonable request.
